# Development of a high resolution melting analysis assay for rapid identification of JAK2 V617F missense mutation and its validation

**DOI:** 10.1186/s40164-019-0134-0

**Published:** 2019-04-30

**Authors:** Alireza Moradabadi, Alireza Farsinejad, Behzad Khansarinejad, Ahamd Fatemi

**Affiliations:** 1 Student Research Committee, Faculty of Allied Medicine, Kerman University of Medical Sciences, Kerman, Iran; 2 Department of Hematology and Medical Laboratory Sciences, Faculty of Allied Medicine, Kerman University of Medical Sciences, Kerman, Iran; 3 Molecular and Medicine Research Center, Arak University of Medical Sciences, Arak, Iran

**Keywords:** JAK2 V617F, ARMS PCR, PCR RFLP, HRM, Diagnosis methods

## Abstract

**Background:**

Myeloproliferative neoplasms (MPN) are heterogeneous diseases that classified by the presence of Philadelphia chromosome into Philadelphia chromosome negative (Ph-neg) and positive (Ph-pos) myeloproliferative neoplasms. In ph-neg group A somatic point mutation (c.1849G>T) in the JAK2 gene, part of the JAK2-STAT signal-transduction pathway, causes substitution of phenylalanine for valine (V617F) in the JAK2 protein and has been identified. This mutation was seen in PV by 65% to 97% and ET (30–57%) and primary myelofibrosis (35–95%). Highly sensitive methods have been used to determine the presence of the JAK2V617F mutation instead of direct sequencing. We aimed to assess JAK2 exon14 mutations by high-resolution melting (HRM) analysis, which allows variation screening in compare to other method for detecting mutation.

**Methods:**

The mutation analysis included 45 individuals who were subjected for diagnosis of ph-neg MPN. Genomic DNA was isolated and different methods are performed.

**Results:**

PCR RFLP, ARMS PCR and HRM method has a detection sensitivity comparable with conventional methods (Qiagen) to identify the mutations and sequencing.

**Conclusions:**

For HRM analysis is cost-effective and beside that it is enzyme independence method also this method able to show amount of the mutant allele carried in samples and it’s helpful for treatments follow-up and determining MRD for them.

## Introduction

Myeloproliferative neoplasms (MPN) are heterogeneous diseases that are characterized by increased pan-cellular production in hematopoietic organs, mainly in bone marrow. The majority of the cells that increase in numbers are non-lymphoid cells and platelets in peripheral blood [1–4]. MPNs are classified by the presence of Philadelphia chromosome into Philadelphia chromosome negative (Ph-neg) and positive (Ph-pos) myeloproliferative neoplasms. A somatic point mutation (c.1849G>T) in the JAK2 gene, part of the JAK2-STAT signal-transduction pathway, causes substitution of phenylalanine for valine (V617F) in the JAK2 protein and has been identified in Ph-neg MPNs especially in polycythemia vera (PV) [1–5].

This mutation is involved in the pathogenesis of PV. The mutation is also present in essential thrombocythemia (ET) and primary myelofibrosis but is not specific for this group of disease and some patients with this disease do not have this mutation. Estimation of the frequency of this mutation has been variable in different studies. Highest percentages were seen in PV by 65% to 97% [6], and the percentage of ET (30–57%) and primary myelofibrosis (35–95%) are slightly lower than the PV cases. The variation of the reported percentages is mainly caused by the sensitivity of the detection method as the higher the sensitivity of the method, the higher the frequency of reported mutations in PV [2–5, 7–10]. The sensitivity of the method should be carefully considered as with using too sensitive methods the rate of false positive results increases, and with using a low sensitivity method there would be increased false negative results [3, 7, 9, 11–13]. Recent molecular methods including ARMS PCR, PCR–RFLP and HRM are highly sensitive and have been used for detection of JAK2 exon 14 (V617F) mutation. Correct diagnosis of this mutation is very important as it is very rare in other similar disorders such as myelodysplasia, acute leukemia and other neoplasms without a history of MPNs. There are also some other mutations in exon 12 of the JAK2 gene that is involved in the pathogenesis of PV. Approximately 3% of PV cases have one of these mutations. In addition, new mutations such as C616Y, D620E, and C618R have been detected in patients with myeloproliferative neoplasms (MPN) [14, 15].

The JAK2 V617F mutation is associated with constitutive activation of the tyrosine kinase in the absence of cytokines, resulting in cell proliferation and survival [1]. Therefore, the JAK2V617F mutation has an important role in the pathogenesis of MPN related disease and also the clinical manifestation of them [5, 16–21].

Highly sensitive methods have been used to determine the presence of the JAK2V617F mutation instead of direct sequencing [3, 7, 9–13, 22]. These methods are ARMS PCR, PCR RFLP, and HRM Real-time PCR. The aim of this study was to develop an HRM method for the detection of JAK2 exon 14 V617F mutation and compare its results with some conventional molecular assays.

## Materials and methods

### Patients

A total number of 45 individuals who were subjected for diagnosis of MPN such as PV and ET, Erythrocytosis or primary non-myeloproliferative Erythrocytosis, and some cases of secondary thrombocytosis, were enrolled in this study. The study approved in Kamran university of medical science ethical committee and The Ethics Approval Code is IR.KMU.REC.1395.812. The peripheral blood samples were obtained from Emam-Reza laboratory, Arak, Iran, between October 2015 and December 2016. The samples of healthy individuals with normal hemoglobin and platelet levels were subjected as the control group. The JAK2 mutational analysis was performed on extracted DNA from whole peripheral blood.

### DNA extraction

Genomic DNA was isolated by using the QIAamp DNA Mini Kit (QIAGEN Germany) based on the manufacturer protocol. The DNA concentration was determined by Nano Drop^®^ at a wavelength of 260, and the absorption ratio of a pure sample DNA at a wavelength of 260 nm to 280 nm was calculated (260/280 ratio).

### HRM analysis of JAK2 exon 14 mutations

Specific primers were designed to amplify the exon 14 mutated region of the JAK2 gene. The designed primers have the following sequence and were synthesized by Tib Molbiol (Berlin, Germany):

HRM. F: 5′-TTGAAGCAGCAAGTATGATG-3′,

HRM. R: 5′CTTACTCTCGTCTCCACAG-3′

HRM Real-time PCR was performed using 10 μl of Type-It Master Mix (QIAGEN, Germany), 2 μl of DNA, 0.7 μl of each of forward and reverse primers (10 pmol) and 6.6 μl of nuclease-free water in a total volume of 20 μl. Thermal cycling conditions included an initial activation step at 95 °C for 5 min followed by 40 cycles including a denaturation step at 94 °C for 20 s and a combined annealing/elongation step at 60 °C for 30 s.

The reaction took place in the LIGHTCYCLER^®^ 96 System (Roche Diagnostics, Mannheim, Germany). For HRM analysis, the PCR products were melted by warming up the temperature from 40 to 95 °C at a ramp rate of 0.007 °C s^−1^ by 20 fluorescence acquisition every degree of temperature. Dissociation of fluorescent dye from double-stranded DNA occurred with an increase in temperature. The normalized graph and the normalized temperature-shifted difference graph (difference graph) from the gene scanning analysis were used to analyze the data. These data come from synthetic DNA samples with 75%, 50%, 25% and 0% allele burden for JAK2V617F mutation (obtained from Bird company, Italy). HRM data were analyzed using the LIGHTCYCLER^®^ 96 software.

### ARMS PCR analysis of JAK2 exon 14 mutations

Primers for the JAK2 gene were designed, one specific for the mutant sequence and the other for the normal sequence. Sequences were as follows:

ARMS. F1: 5′ TGGTTTTATATTATGGAGTATGTT 3′

ARMS. F2: 5′ TGGTTTTATATTATGGAGTATGTG 3′

ARMS. R: 5′ TGGGCATTGTAACCTTCTACT 3′

A segment of the JAK2 gene of about 250 bp was amplified. The forward mutant and normal forward primers were used in separate reactions. ARMS-PCR was performed using 12.5 µl of master mix (amplicon), 1 μl of DNA, 1 μl of each of forward and reverse primers (10 pmol) and 9.5 μl of nuclease-free water in a total volume of 25 μl. Next, the samples were placed in Bio-Rad C1000 Touch Thermal Cycler, programmed in the following cycles: initial denaturation at 95 °C for 5 min, 35 cycles of denaturation at 94 °C for 20 s, annealing at 57 °C for 30 s and primer extension step at 72 °C for 30 s. Ultimately, the final primer extension step was done at 72 °C for 5 min. The samples, including 100 bp marker and positive control, were run on a 2% agarose gel containing 0.1 μg/ml SYBR Safe color.

### PCR RFLP analysis of JAK2 exon 14 mutations

The designed primers to amplify the exon 14 mutated region of the JAK2 gene have the following sequences:

PCR RFLP. F: 5′-AGGACTTTTCTGAGGATACA-3′

PCR RFLP. F: 5′-ATAGTTTACACTGACACCTA-3′

**Fig. 1:**
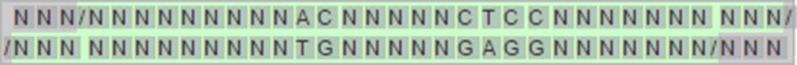
Recognition site of BsaXI enzyme

A segment of the JAK2 gene of about 1147 bp was amplified. PCR–RFLP was performed using 12.5 µl of master mix (amplicon), 1 μl of DNA, 1 μl of each of forward and reverse primers (10 pmol) and 9.5 μl of nuclease-free water in a total volume of 25 μl. Next, the samples were placed in 1000 °C. Touch thermal cycler Bio-Rad, programmed in the following cycle: initial denaturation at 95 °C for 5 min, 35 cycles of denaturation at 94 °C for 20 s, annealing at 52 °C for 30 s and primer extension step at 72 °C for 30 s. Ultimately, the final primer extension step was done at 72 °C for 5 min. A part of the PCR product in association with a 100 bp marker were run on agarose gel 2% containing 0.1 µg/ml SYBR Safe color. After that, the remaining of amplified sample was digested by BsaXI (NEB Cat No: R0609S) restriction enzyme, which cuts the specific site related to the JAK2V617F mutation. Recognition site of the enzyme is shown in Fig. 1. The change of this sequence occurs in the JAK2 V617F mutation with the guanine to thymine substitution at nucleotide 1849 in exon 14 of the JAK2 gene.

### Comparison with standard methods

The test and control samples were analyzed using Ipsogen JAK2 Mutant Kit (Qiagen, Germany), as the reference method.

## Results

### ARMS-PCR results

**Fig. 2:**
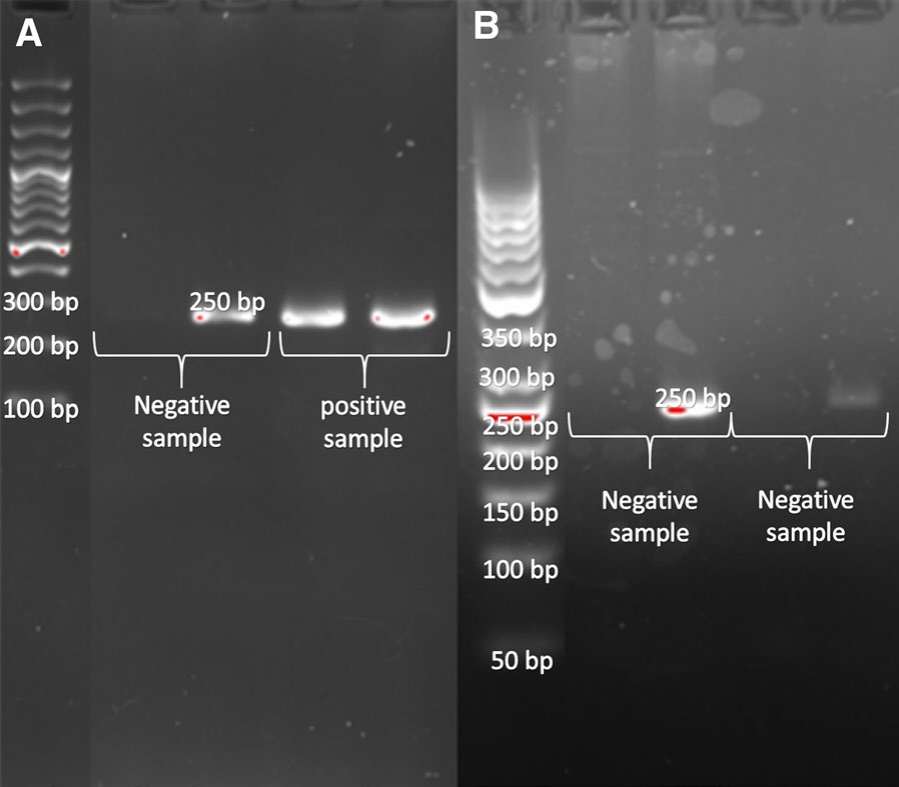
ARMS PCR method (**a**) a negative and heterozygote positive sample, mutant forward primer and wild type forward primer (**b**) negative samples, mutant forward primer had not been amplified and wild type forward primer have been amplified

In the ARMS-PCR assay, the forward mutant-specific primer and reverse primer amplified a 250 bp product and showed a mutated allele in the positive sample. In another tube, the forward wild type-specific primer and reverse primer amplified a 250 bp product showing presence of the wild type allele (Fig. 2). ARMS-PCR Results of JAK2 mutation detection was the same as the standard method in 47 samples. The positive sample shows heterozygote allele burden in this assay. In this assay, the standard positive sample that shows 50% and 75% allele burden subjected and the result show heterozygosity.

### PCR–RFLP results

**Fig. 3:**
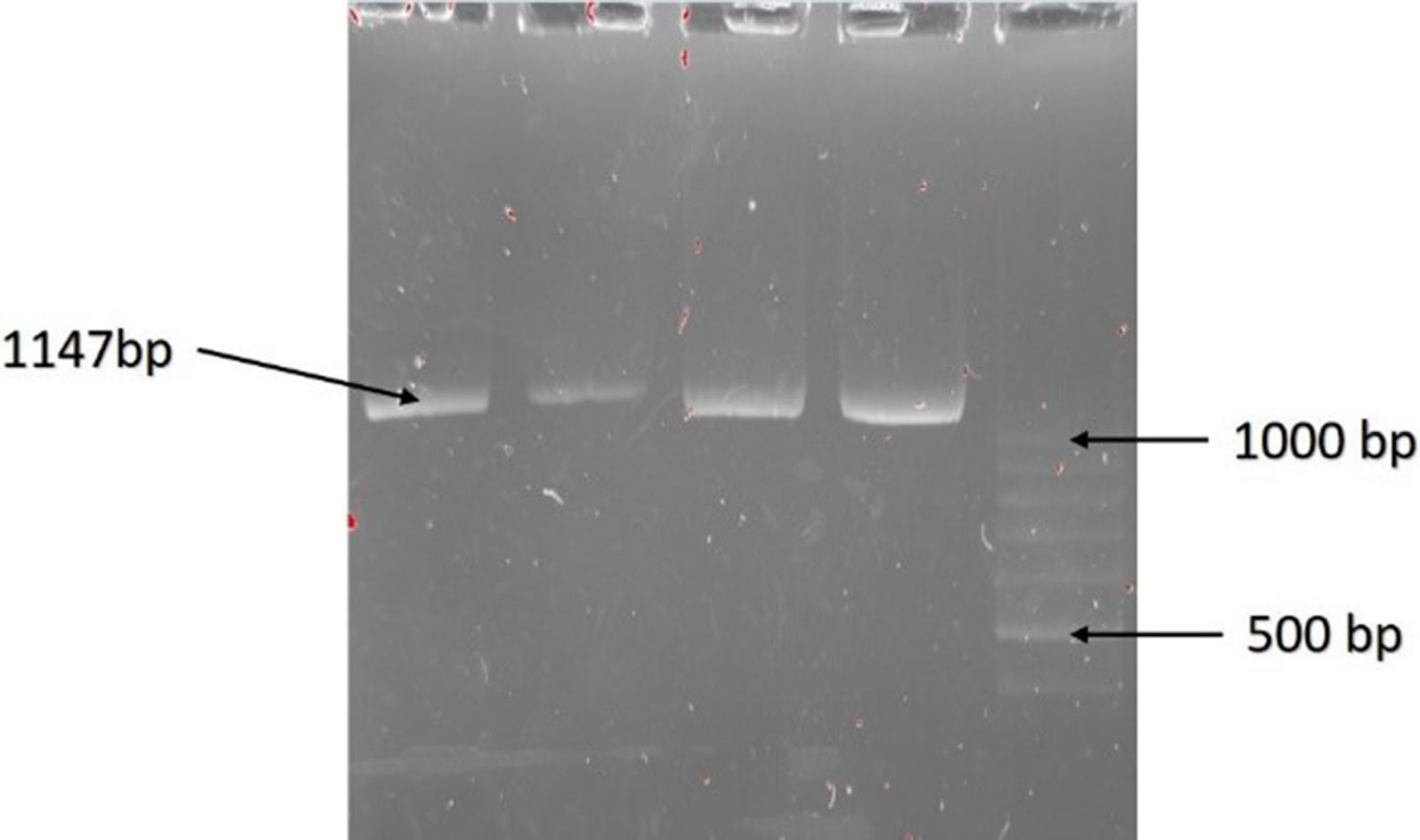
Amplified product before enzyme digestion shows 1147 bp in safe stain gel agarose

**Fig. 4:**
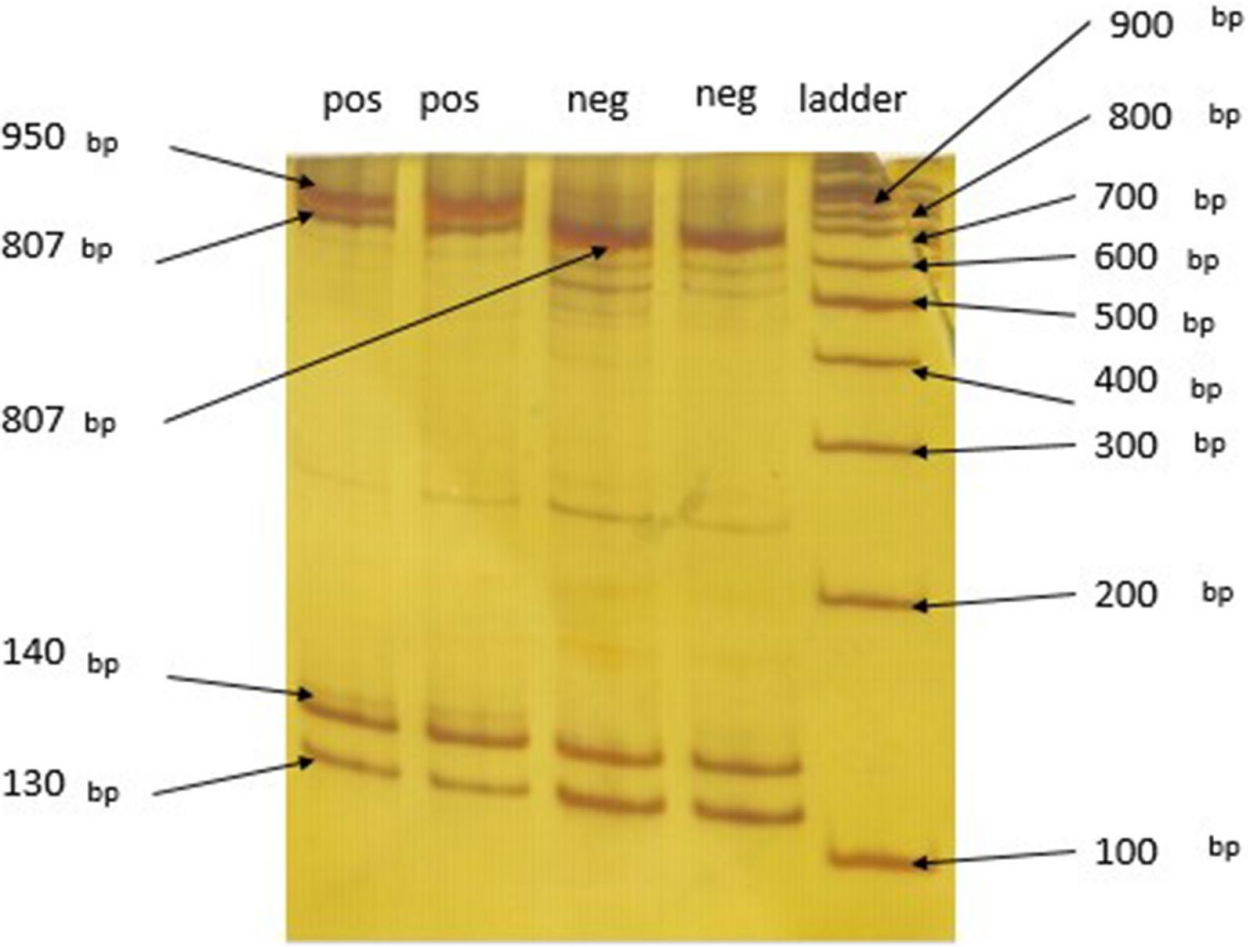
Digested amplified with BsaXI in polyacrylamide gel stained by silver. It shows 950 bp, 807 bp, 140 bp and 130 bp in heterozygote positive rows also 807 bp, 140 bp and 130 bp in negative rows. The ladder is 100 bp

PCR–RFLP requires generation of an amplified product. Forward and reverse primers were used to amplify an 1147 bp product (Fig. 3). Products were then used for cleavage with BsaXI enzyme. With the wild type allele, the 2 recognition sites in the amplified product make 130 bp, 140 bp and 807 bp cleaved products, and also two 30 bp products that are not detected in the gel. In the mutant allele one of the cleavage sites is omitted by the mutation, resulting in absence of the 140 bp product and a new 950 bp product. If a heterozygote sample is digested by BsaXI the 130 bp, 140 bp and 807 bp cleaved products are generated by the wild type allele and the mutant allele gives 130 bp and 950 bp cleaved products. At the end after electrophoresis the 130 bp, 140 bp, 807 bp and 950 bp product are seen in the polyacrylamide gel stained by Silver staining method (Fig. 4).

### Real time PCR and HRM results

**Fig. 5:**
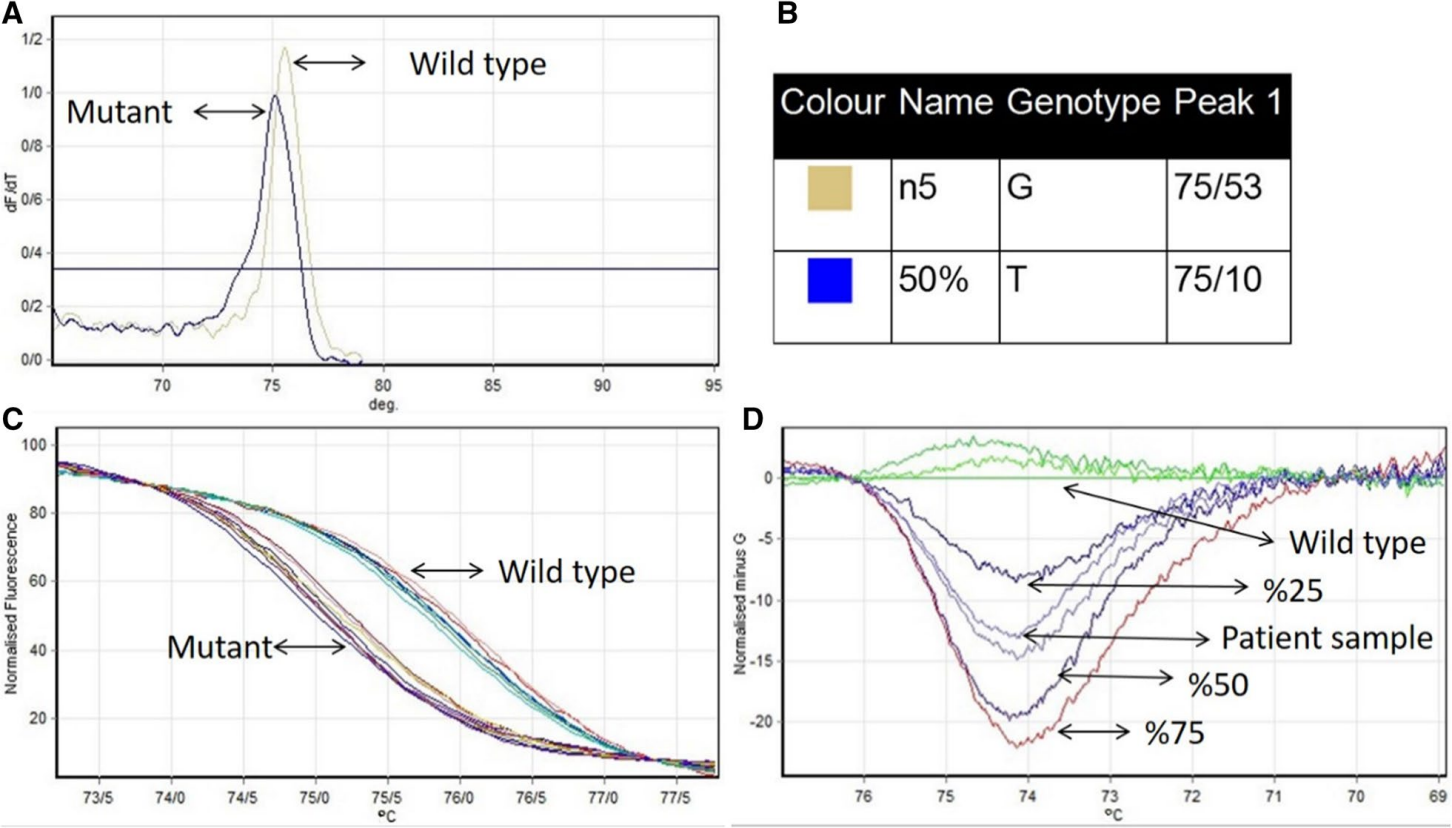
**a**, **b** Melting peak in wild type and mutant sample for the JAK2 V617F mutation shows different temperature (75/53 and 75/10, respectively). **c** The normalized fluorescence plot which synchronization samples in maximum and minimum fluorescence emission. **d** Normalized and temperature-shifted difference plot of the various kinds of allele burden mutation in compare of wild type (G allele)

Primer pairs of HRM amplified an 80 bp product, whether the sample was positive or negative for the JAK2V617F mutation (c.1849G>T). In HRM analysis, positive and negative sample have different melting peaks (Fig. 5a, b). This shows c.1849G>T replacement caused a decrease in melting peak. To further differentiate the mutant and wild type, a high-resolution difference plot of the HRM assay was plotted by subtracting the melting curve of each species from the baseline (or reference) curve. The baseline curve shape is constructed by negative sample and 0% synthetic DNA for JAK2 V617F. Based on the patterns of the difference plots, the assay was able to discriminate JAK2 V617F mutant allele from wild type and also could differentiate allele burden in the sample by the distance of different melt curves from the baseline (Fig. 5d).

### Sensitivity and specificity of methods

The sensitivity and specificity of Ipsogen JAK2 Mutant Kit (Qiagen, Germany) confirmed by sequencing, HRM, PCR–RFLP and, ARMS PCR methods, calculate in 45 cases were 20 and 24 cases were negative and positive, respectively, the positive and negative cases confirmed by Ipsogen JAK2 Mutant Kit (Qiagen, Germany) confirmed by sequencing as gold standard method. It means all positive sequenced samples were also positive in other methods, like negative cases. These data are summarized in Table 1.

**Table 1:** Sensitivity and specificity of Ipsogen JAK2 Mutant Kit (Qiagen, Germany) confirmed by sequencing, HRM, PCR–RFLP and, ARMS PCR methods

Sample	Mutant		Wild type		Sensitivity (%)	Specificity (%)
	No. tests	Positive	No. tests	Negative		
Ipsogen JAK2 mutant Kit (Qiagen, Germany) confirmed by sequencing	25	25	20	20	100	100
ARMS	25	25	20	20	100	100
PCR–RFLP	25	25	20	20	100	100
HRM	25	25	20	20	100	100

## Discussion

Myeloproliferative neoplasms (MPN) are the heterogeneous disease which classified by the existence of Philadelphia chromosome. In patients with negative Philadelphia chromosome(ph-neg), there is a somatic mutation (c.1849G>T) in JAK2 gene which constitutively activate JAK2-STAT signal-transduction pathway. Detection of this point mutation is critical in diagnosis and treatment of the patients.

In the present study, Results of JAK2 V617F Mutation detection by PCR–RFLP, ARMS-PCR and HRM methods were similar to gold standard methods including Real Time PCR and sequencing. In comparison, each of these methods had some advantages and disadvantages.

The advantages and disadvantages of each tests are different due to the difference between the basis of each method, ARMS-PCR depend on primers and the set up performance, PCR–RFLP depend on the enzyme and the control sequence and the HRM depend on the instrument and the ability of the laboratory to set up the method. The sequencing is gold standard method in diagnosis of mutation Lin and et al. [23] find the Limit of detection (mutant concentration in %) for AS-PCR and HRM 2.5 and 6% of the whole DNA, respectively. In our study the HRM, PCR–RFLP and, ARMS-PCR performed and the HRM can find the allele burden as 25, 50,75%, less than, more them or between them. On the other hand, both PCR–RFLP and ARMS- PCR methods depend on operator and its set up. HRM analysis is an efficient and sensitive PCR–based approach for determining the gene mutation with capability to differentiate heterozygous and homozygous mutations. Furthermore, this method provides the possibility of allele burden measurement in clinical evaluations.

Hong-Cui Cao et al. compared the different approaches to identify JAK2V617F mutations and introduced PCR–RFLP method as a definite and appropriate way to detect this mutation. Also they use the ARMS PCR and other molecular methods to detect mutations and selected sanger sequencing as reference method to identify the mutation. In our study, a modified PCR–RFLP could recognize the presence of mutations in patients as well as the sequencing method [24]. In another study by Tze-Kiong Er and colleagues in order to identify the JAK2 V617F mutation, PCR RFLP method was considered as a reference method for the approval of other methods. They have introduced PCR RFLP as an appropriate but operator-dependent method to identify this mutation [25]. In present study, PCR RFLP method has a detection sensitivity comparable with conventional methods (Qiagen) to identify the mutations and sequencing. Advantages of the present modified PCR–RFLP method include the presence of internal controls to verify the performance of the enzyme cleavages as well as being cost-effective and affordable compared to the sequencing method and other methods such as HRM, which need to special equipment. However, this method is only capable of detecting just homozygous and hetero-zygote causing states. Since the DNA samples were extracted from peripheral blood and this mutation is not present in lymphoid cells, all the samples show the heterozygous state. In a study by Amy Jones et al. to evaluate the methods of identifying mutations in JAK2 V617F, techniques such as ARMS PCR were introduced as an appropriate and cost-effective method in order to examine the exact mutation [7]. Results obtained by the mentioned method in our study were the same as using PCR RFLP and sequencing techniques. The present ARMS PCR method as well as the PCR–RFLP is cost-effective and beside that it is enzyme independence method. This method’s drawback could be that can only detect homozygous and hetero-zygote as well as PCR RFLP. In a study by Hong-Cui Cao et al. HRM method was identified as an easy, sensitive and reliable way to detect JAK2V617F mutation. Since this technique was performed using REAL TIME PCR and Reaction was conducted in a closed tube, contamination error is minimized. Another benefit of this method of identification is about the amount of the mutant allele carried in samples taken from patients, means somehow allele burden, that makes doctors able to not only recognize homozygous and heterozygous states but also to determine the percentage of mutations. This advantage allows the physician to examine the amount of carried mutant allele during and after treatment as well as in the detection of minimal residual disease (MRD). According to a study by Serge Carillo and his colleagues in which the mutation detection rate reaches up to 1% of mutant DNA in samples carrying the mutant allele, the NESSTED HRM method has been used to identify mutations and had a sensitivity of 100% and specificity of 96.7%.

In conclusion, given the particular sensitivity of HRM and many advantages in identifying JAK2V617F mutations, it can be a good way to identify mutations in patients with suspected myeloproliferative diseases using DNA extracted from the peripheral blood. In addition, due to the sensitivity of this method it can be useful in following the patients’ response to the treatment and determining MRD for them.

## References

[1] James C, Ugo V, Le Couedic JP (2005). A unique clonal JAK2 mutation leading to constitutive signalling causes polycythaemia vera. Nature.

[2] Kralovics R, Passamonti F, Buser AS (2005). A gain-of-function mutation of JAK2 in myeloproliferative disorders. N Engl J Med.

[3] Levine RL, Wadleigh M, Cools J (2005). Activating mutation in the tyrosine kinase JAK2 in polycythemia vera, essential thrombocythemia, and myeloid metaplasia with myelofibrosis. Cancer Cell.

[4] Baxter EJ, Scott LM, Campbell PJ (2005). Acquired mutation of the tyrosine kinase JAK2 in human myeloproliferative disorders. Lancet.

[5] Tefferi A, Thiele J, Vardiman JW (2009). The 2008 World Health Organization classification system for myeloproliferative neoplasms. Cancer.

[6] Murugesan G, Aboudola S, Szpurka H (2006). Identification of the JAK2 V617F mutation in chronic myeloproliferative disorders using FRET probes and melting curve analysis. Am J Clin Pathol.

[7] Jones AV, Kreil S, Zoi K (2005). Widespread occurrence of the JAK2 V617F mutation in chronic myeloproliferative disorders. Blood.

[8] Jelinek J, Oki Y, Gharibyan V (2005). JAK2 mutation 1849G>T is rare in acute leukemias but can be found in CMML, Philadelphia chromosome–negative CML, and megakaryocytic leukemia. Blood..

[9] Zhao R, Xing S, Li Z (2005). Identification of an acquired JAK2 mutation in polycythemia vera. J Biol Chem.

[10] Wolanskyj AP, Lasho TL, Schwager SM (2005). JAK2V617F mutation in essential thrombocythemia: clinical associations and long-term prognostic relevance. Br J Haematol.

[11] Poodt J, Fijnheer R, Walsh I (2006). A sensitive and reliable semi-quantitative real-time PCR assay to detect JAK2 V617F in blood. Hematol Oncol.

[12] Rapado I, Albizua E, Ayala R (2008). Validity test study of JAK2 V617F and allele burden quantification in the diagnosis of myeloproliferative diseases. Ann Hematol.

[13] Sidon P, El Housni H, Dessars B (2006). The JAK2V617F mutation is detectable at very low level in peripheral blood of healthy donors. Leukemia.

[14] Grünebach F, Bross-Bach U, Kanz L (2006). Detection of a new JAK2 D620E mutation in addition to V617F in a patient with polycythemia vera. Leukemia.

[15] Schnittger S, Bacher U, Kern W (2006). Report on two novel nucleotide exchanges in the JAK2 pseudokinase domain: D620E and E627E. Leukemia.

[16] Fröhling S, Lipka DB, Kayser S (2006). Rare occurrence of the JAK2 V617F mutation in AML subtypes M5, M6, and M7. Blood.

[17] Pesu M, O’Shea J, Hennighausen L (2005). Identification of an acquired mutation in Jak2 provides molecular insights into the pathogenesis of myeloproliferative disorders. Mol Interventions.

[18] Fröhling S, Scholl C, Gilliland DG (2005). Genetics of myeloid malignancies: pathogenetic and clinical implications. J Clin Oncol.

[19] Antonioli E, Guglielmelli P, Pancrazzi A (2005). Clinical implications of the JAK2 V617F mutation in essential thrombocythemia. Leukemia.

[20] Cazzola M, Skoda R (2005). Gain of function, loss of control-a molecular basis for chronic myeloproliferative disorders. Haematologica.

[21] Tefferi A, Gilliland DG (2005). JAK2 in myeloproliferative disorders is not just another kinase. Cell Cycle.

[22] Link-Lenczowska D, Pallisgaard N, Cordua S (2018). A comparison of qPCR and ddPCR used for quantification of the JAK2 V617F allele burden in Ph negative MPNs. Ann Hematol.

[23] Lin CY, Ho CM, Tamamyan G (2016). Validating the sensitivity of high-resolution melting analysis for JAK2 V617F mutation in the clinical setting. J Clin Lab Anal.

[24] Cao A, Galanello R (2010). Beta-thalassemia. Genet Med.

[25] Er T-K, Lin S-F, Chang J-G (2009). Detection of the JAK2 V617F missense mutation by high resolution melting analysis and its validation. Clin Chim Acta.

